# Interactive Conversational Agents for Health Promotion, Prevention, and Care: Protocol for a Mixed Methods Systematic Scoping Review

**DOI:** 10.2196/40265

**Published:** 2022-10-11

**Authors:** Maxime Sasseville, Romina H Barony Sanchez, Achille R Yameogo, Laurie-Ann Bergeron-Drolet, Frédéric Bergeron, Marie-Pierre Gagnon

**Affiliations:** 1 Faculté des Sciences Infirmières Université Laval Québec, QC Canada; 2 Faculté de Médecine Université Laval Québec, QC Canada; 3 Bibliothèque – Direction des Services-Conseils Université Laval Québec, QC Canada

**Keywords:** conversational agents, chatbots, scoping review, literature review, healthcare, health care, health promotion, prevention, care, computer, natural language processing, literature, community

## Abstract

**Background:**

Interactive conversational agents, also known as “chatbots,” are computer programs that use natural language processing to engage in conversations with humans to provide or collect information. Although the literature on the development and use of chatbots for health interventions is growing, important knowledge gaps remain, such as identifying design aspects relevant to health care and functions to offer transparency in decision-making automation.

**Objective:**

This paper presents the protocol for a scoping review that aims to identify and categorize the interactive conversational agents currently used in health care.

**Methods:**

A mixed methods systematic scoping review will be conducted according to the Arksey and O’Malley framework and the guidance of Peters et al for systematic scoping reviews. A specific search strategy will be formulated for 5 of the most relevant databases to identify studies published in the last 20 years. Two reviewers will independently apply the inclusion criteria using the full texts and extract data. We will use structured narrative summaries of main themes to present a portrait of the current scope of available interactive conversational agents targeting health promotion, prevention, and care. We will also summarize the differences and similarities between these conversational agents.

**Results:**

The search strategy and screening steps were completed in March 2022. Data extraction and analysis started in May 2022, and the results are expected to be published in October 2022.

**Conclusions:**

This fundamental knowledge will be useful for the development of interactive conversational agents adapted to specific groups in vulnerable situations in health care and community settings.

**International Registered Report Identifier (IRRID):**

DERR1-10.2196/40265

## Introduction

Digital technologies are an engine of transformation in the health sector and represent a promising avenue for improving access to care, care personalization, health prevention, and health promotion, particularly for those in vulnerable situations [1]. Health technologies are increasingly necessary to support and improve health care access [2,3].

Conversational agents, also known as “chatbots,” are computer programs that use natural language processing to engage in conversations with humans to provide or collect information [4-6]. Chatbots use algorithms to simulate a human conversation via text or voice messages. They are easy to use for patients and require minimal computer literacy and knowledge [7,8]. Chatbots can use simple predetermined conversation algorithms to simulate conversation or more complex systems based on neural networks and deep learning to understand speech, produce a voiced answer, and simulate social interaction [9]. Chatbots allow for automated interventions with customizable, accessible, and cost-effective software [8].

Although the literature on the development and use of chatbots for patients’ health is growing, there are still important knowledge gaps that remain, such as identifying design aspects relevant to health care and functions to offer transparency in decision-making automation [7,10,11]. Moreover, despite increasing developments in digital technologies and the implementation of conversational agents in a wide range of domains, their use in health care is still limited [12]. Chatbots are primarily developed for a specific context of care, so little is known about the greater contextual application of chatbots in health care. Providing an exhaustive portrait of current chatbots used in health care is a significant step toward understanding the position of chatbots in the current infrastructure of care. This review is interested in chatbots that were able to adapt and simulate interactive conversations as opposed to chatbots offering users constrained multiple-choice options, thus limiting the impression of real and meaningful interactions.

The goal of this mixed methods systematic scoping review is to identify and categorize interactive conversational technologies currently used in health promotion, prevention, and care. 

## Methods

### Overview

A mixed methods systematic scoping review will be conducted according to the Arksey and O’Malley [13] framework and the guidance of Peters et al [14] for systematic scoping reviews. This literature review will focus on studies on interactive conversational agents for health promotion, prevention, and care. As this topic is complex and heterogeneous, a review of the scope of the literature will help tailor future systematic reviews and research endeavors. The PRISMA-ScR (Preferred Reporting Items for Systematic Reviews and Meta-Analyses Extension for Scoping Reviews) criteria will be used to report the results [15].

Our key synthesis question is as follows: (1) in which population and settings are interactive conversational agents used for health promotion, prevention, and care? and (2) what are the characteristics of the technologies supporting these interactive conversational agents?

### Eligibility Criteria

We will address all types of evidence matching the PICOS (population, intervention, comparison, outcomes, setting or context) criteria:

- Participants or population: we will include all studies targeting laypeople (eg, patients, students, citizens). Studies targeting only health care providers will be excluded.
- Interventions or phenomena of interest: we will include all interactive conversational agents, that is, those involving a 2-way exchange (written, oral, and visual dialogue), directed at laypeople for health promotion, prevention, and care.
- Comparator: no restrictions will be applied.
- Outcomes: we will consider all outcomes reported in the studies. We will seek outcomes related to patients, caregivers, health care providers, and policy makers. Those could include barriers, facilitators, acceptability, feasibility, adoption, fidelity, morbidity, mortality, quality of life, satisfaction, cost, and cost-effectiveness.
- Setting: we will include studies taking place in a health care or community setting, in any geographical area. All types of studies will be included (qualitative, quantitative, and mixed methods).

### Search Strategy

The search strategy was developed in collaboration with a university librarian who has experience in literature reviews (FB) (Multimedia Appendix 1). An iterative process of revision by members of the research team took place, and all relevant comments were integrated into the final version of the search strategy. The final version was approved by all team members. A specific search strategy was formulated for each of the following databases: MEDLINE (Ovid), Embase, Inspec (Engineering Village), Web of Science, and CINAHL. The search will be restricted to studies from the last 20 years because of the recent nature of conversational agents. No restriction on language was applied to the search. Terms such as *chatbot*, *conversational agent*, *virtual embodied agent*, and their spelling variants were used for the search. Additionally, since innovation implementations are often conducted by the private sector, we performed an internet search in the following sources and databases: Google, Google Scholar, Institut national d’excellence en santé et en services sociaux, Canadian Evaluation Society, EuroScan, OpenGrey, Grey Literature Report, GreyNet, and Grey Matters. The search also included a hand search, and bibliographies were reviewed for additional relevant references. The search strategy is presented in Multimedia Appendix 1.

### Data Collection

All citations will be exported to the online collaboration tool Covidence (Veritas Health Innovation) [16], where duplicates will be removed by its automated function. An independent assessment of inclusion criteria will be done by at least 2 reviewers. Reviewers will search and obtain all the full texts of the selected references and will import the PDF files in Covidence. Two reviewers will independently apply the inclusion criteria using the full texts. Reasons for exclusion will be recorded in Covidence. A PRISMA flowchart will be used to describe the identifications of studies, the screening process, and the application of inclusion and exclusion criteria [17].

### Data Extraction

Team members will complete the extraction, and the data will be reviewed by an experienced researcher (MPG or MS). We will extract descriptive data (title, year of publication, authors, country), study type (published or gray literature, study design), intervention data (name of the technology, language of the technology, implementation channel, and features like audio, text, voice, avatar), setting data (target population, place of implementation), sample data (comparator, number of participants, sample sizes), outcomes (process, patients, providers, or systems related), and outcome type (qualitative and quantitative). We will appraise the quality of the studies included by applying the Mixed Methods Appraisal Tool [18].

### Data Synthesis

We will complete the data synthesis using structured narrative summaries of main themes and provide a portrait of the current scope of available technologies using descriptive analysis. A summary of the differences and similarities between conversational agents will highlight the following points: strengths and weaknesses, main outcomes, main resources used, and trade-offs. A map of outcomes and targeted populations will be built and presented [14].

## Results

The search strategy and screening steps of the review were completed in March 2022. Data extraction and analysis began in May 2022, and the results are expected to be published in October 2022.

## Discussion

### Main Contributions of This Scoping Review

Literature reviews on chatbots in health care are available in specific contexts, such as mental health management [19-21], prevention of COVID-19 [22], health behavior change [23,24], chronic conditions management [25], and medical education [26]. A systematic review by Laranjo et al [12] was completed in 2018 with a global scope, but it was aimed at patients and providers and focused only on verbal communication. To our knowledge, our review is the first endeavor aiming to offer an overarching map of the research on interactive conversational agents targeting laypeople in all contexts of care. As there are many pilot and usability studies on the topic, summarizing evidence by context and type of technology will offer a unique view of how and for whom conversational agents are used. This review will also explore whether these technologies are useful and successful in managing health issues.

### Potential Impact and Future Directions

There is a human resources crisis in health care. Worldwide shortages in health care professionals, increasing burnout rates, and an aging population are 3 major factors causing an imbalance in the offering and demand of health care services [27,28]. Focused technologies and tools such as interactive conversational agents are needed to overcome the potential abyss of human resource shortage in health care by reducing cost and improving access. Understanding what is currently offered in health care has the potential to help researchers, health care professionals, and decision-makers to develop, implement, or test solutions.

Accelerated by the COVID-19 pandemic, the shift to digital care has caused health inequities [29]. This review will thus pay particular attention to how equity, diversity, and inclusion have been considered in the development of conversational agents. Vulnerable populations often do not have the skills and opportunities to use chatbots [30]. Indeed, personal characteristics (eg, advanced age, chronic diseases, disability situation, lack of digital literacy) or contextual factors (eg, poverty, housing, irregular migrant status) may make chatbots unfavorable for use. Moreover, in their interaction with individuals, chatbots cannot always evolve with time and adapt to the user’s literacy skills and language level. Thus, they sometimes are unable to adapt to dynamic user behavior and offer customized responses tailored to the user’s personality [31,32]. Therefore, the development of conversational agents adapted to the needs of individuals, especially those in vulnerable situations, remains a major challenge for researchers [33]. This review could contribute to the inclusion of the needs of vulnerable populations when designing conversational agents [30].

Finally, the widespread implementation of conversational agents is still burdened by ethical and clinical implications [23]. Shifting to an artificial relationship removes human-to-human communication from the care process. Understanding the level of involvement of professionals in the human-machine relationship remains an area of research worth considering. The burden of decision-making also emanates from that problem. As professionals are assisted by technological tools, we need to think about who is responsible for the information and decisions offered by technology that can be potentially harmful to patients [34]. On a more macro level, the use of conversational agents could support a shift toward disease prevention rather than treatment, thus leading to large-scale benefits for the whole society [7].

### Conclusion

The results of this scoping review will provide a portrait of interactive conversational agents and their various applications in health promotion, prevention, and care. This fundamental knowledge will be useful for the development of interactive conversational agents adapted to specific users and people in vulnerable situations.

## Appendix

Multimedia Appendix 1 Detailed search strategy.

## Abbreviations

PRISMA: Preferred Reporting Items for Systematic Reviews and Meta-Analyses
PRISMA-ScR: Preferred Reporting Items for Systematic Reviews and Meta-Analyses Extension for Scoping Reviews
PICOS: population, intervention, comparison, outcomes, setting or context

## Data Availability

The data for this review will be deposited and made available online in the Open Science Framework depository.
