# Supplementary material for: Interactive Conversational Agents for Health Promotion, Prevention, and Care: Protocol for a Mixed Methods Systematic Scoping Review
Source: JMIR Res Protoc. 2022 Oct 11;11(10):e40265. doi: 10.2196/40265 (PMC9597423; doi:10.2196/40265)
Supplement: Multimedia Appendix 1 [file resprot_v11i10e40265_app1.pdf]

Medline (OVID)

Date of the search:

Database limit: No database limit has been apply

#	Search strategy	Results
1	("chat bot?" or chatterbot? or chatbot? or medbot? or "chatter bot?" or smart bot? or smartbot?).ti,ab,kw	290
2	(Conversational adj2 (host or coach or avatar or advisor or assistant or interface or avatar or agent? or system or computer or humanoid or character or bot? or AI)).ti,ab	240
3	((virtual or intelligent or chat or computer or AI or "artificial intelligence" or relational or embodied) adj2 agent?).ti,ab	805
4	1 or 2 or 3	1192

Embase (Embase.com)

Date of the search:

Database limit: Embase results only

#	Search strategy	Results
1	("chat bot$" OR chatterbot$ OR chatbot$ OR medbot$ OR "chatter bot$" OR "smart bot$" OR smartbot$):ti,ab,kw	32
2	(Conversational NEAR/2 (host OR coach OR avatar OR advisor OR assistant OR interface OR avatar OR agent$ OR system OR computer OR humanoid OR character OR bot$ OR AI)):ti,ab	252
3	((virtual OR intelligent OR chat OR computer OR AI OR "artificial intelligence" OR relational OR embodied) NEAR/2 agent$):ti,ab	853
4	#1 or #2 or #3	1,300
5		420

CINAHL

Date of the search:

Database limit: No database limit has been apply

#	Search strategy	Results
1	TI ("chat bot?" OR chatterbot? OR chatbot? OR medbot? OR "chatter bot?" OR smart bot? OR smartbot?) OR AB ("chat bot?" OR chatterbot? OR chatbot? OR medbot? OR "chatter bot?" OR smart bot? OR smartbot?)	195
2	TI (Conversational N2 (host OR coach OR avatar OR advisor OR assistant OR interface OR avatar OR agent? OR system OR computer OR humanoid OR character OR bot? OR AI)) OR AB (Conversational N2 (host OR coach OR avatar OR advisor OR assistant OR interface OR avatar OR agent? OR system OR computer OR humanoid OR character OR bot? OR AI))	150
3	TI ((virtual OR intelligent OR chat OR computer OR AI OR "artificial intelligence" OR relational OR embodied) N2 agent?) OR AB ((virtual OR intelligent OR chat OR computer OR AI OR "artificial intelligence" OR relational OR embodied) N2 agent?)	258
4	S1 or S2 or S3	517

Web of Science

Date of the search:

Database limit: limit results to articles only

#	Search strategy	Results
1	TS=("chat bot$" OR chatterbot$ OR chatbot$ OR medbot$ OR "chatter bot$" OR smart bot$ OR smartbot$)	34,228
2	TS=(Conversational NEAR/2 (host OR coach OR avatar OR advisor OR assistant OR interface OR avatar OR agent$ OR system OR computer OR humanoid OR character OR bot$ OR AI))	3,014
3	TS=((virtual OR intelligent OR chat OR computer OR AI OR "artificial intelligence" OR relational OR embodied) NEAR/2 agent$)	14,984
4	#1 OR #2 OR #3	50,662
5	TS=(patient)	6,981,610
6	#4 AND #5	2,042
7	Articles database limits	1,330

Inspec (Engineering Village)
Date of the search:
Database limit: limit results to articles only

#	Search strategy	Results
1	chatbots WN CV	180
2	"chat bot*" WN TI OR chatterbot* WN TI OR chatbot* WN TI OR medbot* WN TI OR "chatter bot*" WN TI OR smart bot* WN TI OR smartbot* WN TI OR "chat bot*" WN AU OR chatterbot* WN AU OR chatbot* WN AU OR medbot* WN AU OR "chatter bot*" WN AU OR smart bot* WN AU OR smartbot* WN AU	929
3	(Conversational NEAR/2 host) WN TI OR (Conversational NEAR/2 coach) WN TI OR (Conversational NEAR/2 avatar) WN TI OR (Conversational NEAR/2 advisor) WN TI OR (Conversational NEAR/2 assistant) WN TI OR (Conversational NEAR/2 interface) WN TI OR (Conversational NEAR/2 avatar) WN TI OR (Conversational NEAR/2 agent*) WN TI OR (Conversational NEAR/2 system) WN TI OR (Conversational NEAR/2 computer) WN TI OR (Conversational NEAR/2 humanoid) WN TI OR (Conversational NEAR/2 character) WN TI OR (Conversational NEAR/2 bot*) WN TI OR (Conversational NEAR/2 AI) WN TI OR (Conversational NEAR/2 host) WN AU OR (Conversational NEAR/2 coach) WN AU OR (Conversational NEAR/2 avatar) WN AU OR (Conversational NEAR/2 advisor) WN AU OR (Conversational NEAR/2 assistant) WN AU OR (Conversational NEAR/2 interface) WN AU OR (Conversational NEAR/2 avatar) WN AU OR (Conversational NEAR/2 agent*) WN AU OR (Conversational NEAR/2 system) WN AU OR (Conversational NEAR/2 computer) WN AU OR (Conversational NEAR/2 humanoid) WN AU OR (Conversational NEAR/2 character) WN AU OR (Conversational NEAR/2 bot*) WN AU OR (Conversational NEAR/2 AI) WN AU	926
4	(virtual NEAR/2 agent) WN TI OR (intelligent NEAR/2 agent) WN TI OR (chat NEAR/2 agent) WN TI OR (computer NEAR/2 agent) WN TI OR (AI NEAR/2 agent) WN TI OR ("artificial intelligence" NEAR/2 agent) WN TI OR (relational NEAR/2 agent) WN TI OR (embodied NEAR/2 agent) WN TI OR (virtual NEAR/2 agent) WN AU OR (intelligent NEAR/2 agent) WN AU OR (chat NEAR/2 agent) WN AU OR (computer NEAR/2 agent) WN AU OR (AI NEAR/2 agent) WN AU OR ("artificial intelligence" NEAR/2 agent) WN AU OR (relational NEAR/2 agent) WN AU OR (embodied NEAR/2 agent) WN AU	1,984
5	#1 OR #2 OR #3 OR #4	3,797

6	patient WN TI OR patient WN AU	12,765
7	#5 AND #6	7

Search results – Custom Google search engines

<u>Custom Google Search Engine for Government Documents</u>
Note: filter was applied to limit search to Canadian documents.
Date searched: 30-Mar-15
Searches "All results" – first 10 pages, representing 1000 results screened

#	Search	# results	# results screened	# new potentially relevant records	Total # records
1	school AND breakfast meal snack milk AND guidelines OR policies	~ 476,000	100	18	18
2	meals OR nutrition OR feeding AND school AND programs	~ 42,200,000	100	3	21
3	school nutrition AND policies or guidelines	~ 4,210,000	100	4	25
4	school meals AND best practices	~ 4,000,000	100	0	25
5	nutrition guidelines AND school program	~ 3,700,000	100	0	25
6	breakfast AND school AND recommendations or guidelines	~ 4,080,000	100	5	30
7	milk program AND school	~ 7,700,000	100	2	32
8	fruits vegetables AND school AND program OR initiative	~ 2,790,000	100	3	35
9	school AND meal OR breakfast AND guidelines OR policies	~ 13,700,000	100	0	35
10	meal program AND school	~ 22,100,000	100	0	35

<u>Custom Google Search Engine for Canadian Public Health Information</u>
Date searched: 31-Mar-15
Searches "All results" – first 10 pages, representing 1000 results screened

#	Search	# results	# results screened	# new potentially relevant records	Total # records
1	school AND breakfast meal snack milk AND guidelines OR policies	~ 466,000	100	4	39
2	meals OR nutrition OR feeding AND school AND programs	~ 36,700,000	100	2	41
3	school nutrition AND policies or guidelines	~ 4,180,000	100	2	43
4	school meals AND best practices	~ 3,850,000	100	0	43
5	nutrition guidelines AND school program	~ 3,690,000	100	2	45

6	breakfast AND school AND recommendations or guidelines	~ 4,850,000	100	0	45
7	milk program AND school	~ 7,730,000	100	1	46
8	fruits vegetables AND school AND program OR initiative	~ 2,520,000	100	0	46
9	school AND meal OR breakfast AND guidelines OR policies	~ 16,100,000	100	1	47
10	meal program AND school	~ 21,100,000	100	0	47

Eureka : 95 results

LEAD= "Chatbot"

| LEAD= ("agent conversationnel")

| LEAD= ("agent virtuel")

| LEAD= ("virtual agent")

| LEAD= ("virtual assistant")

| LEAD= ("chat bot")

| LEAD= ("chatterbot")

& LEAD= Health| santé
